# Intraoperative Findings of Inferior Petrosal Vein During Microvascular Decompression for Hemifacial Spasm: A Single-Surgeon Experience

**DOI:** 10.3389/fsurg.2022.921589

**Published:** 2022-06-10

**Authors:** Mengyang Wang, Jiajing Wang, Xiuling Zhang, Songshan Chai, Yuankun Cai, Xuan Dai, Bangkun Yang, Wen Liu, Taojunjin Lu, Zhimin Mei, Zhixin Zheng, YiXuan Zhou, Jingyi Yang, Lei Shen, Jingwei Zhao, Joshua Ho, Meng Cai, Jincao Chen, Nanxiang Xiong

**Affiliations:** ^1^ Department of Neurosurgery, Zhongnan Hospital of Wuhan University, Wuhan, China; ^2^ Department of Neurosurgery, Union Hospital, Tongji Medical College, Huazhong University of Science and Technology, Wuhan, China; ^3^ Department of Neurology, Xiaogan Hospital Affiliated to Wuhan University of Science and Technology, Xiaogan, China; ^4^ School of Biomedical Sciences, LKS Faculty of Medicine, Hongkong University, Hongkong, China; ^5^ iRegene Therapeutics Ltd., Wuhan, Hongkong, China

**Keywords:** microvascular decompression, inferior petrosal vein, hemifacial spasm, anatomy, intraoperative decisionmaking

## Abstract

**Objective:**

This study aims to evaluate the impact of the inferior petrosal veins (IPVs) on operational exploration and to analyze related anatomic features.

**Methods:**

A total of 317 patients were retrospectively studied. Surgical outcomes and postoperative complications were analyzed, and patients were divided into two groups according to whether the IPV was sacrificed or preserved. The diameter of the IPV was also recorded during operation. Furthermore, the position where the IPV drained into the jugular bulb was recorded in each patient, and the influence of different injection points on the operation was analyzed.

**Results:**

IPVs were conclusively identified in 242/317 (76.3%) of patients, with 110/242 (45.5%) of patients categorized as “IPV sacrifice” versus 132/242 (54.5%) categorized as “IPV preservation.” IPV diameter was observed to be <0.5 mm in 58 cases (23.9%), 0.5 mm–1.0 mm (≥0.5 mm and ≤1.0 mm) in 145 cases (59.9%), and >1 mm in 39 cases (16.2%). The position of IPV drainage into the jugular bulb was at the level of the accessory nerve in 163 cases (67.3%), the level of the vagus nerve in 42 cases (17.4%), and the level of the glossopharyngeal nerve or above in 37 cases (15.3%). The diameters of IPV in the sacrifice group were mainly less than 1 mm (94.5% vs. 75%, *P *< 0.01), and the cases with draining points near the glossopharyngeal nerve were more than that in the preservation group (27.3% vs. 5.3%, *P* < 0.01).

**Conclusion:**

IPV is an obstructive structure in MVD for HFS, with considerable variations in diameters and draining points. IPV near the glossopharyngeal nerve significantly impacts surgical exposure and is often sacrificed for a better view of the operation field. Meanwhile, it is feasible to maintain IPVs with a diameter >1 mm.

## Introduction

In recent years, the clinical management and outcomes of Microvascular decompression (MVD) surgery have significantly improved (1, 2). These developments, while undeniably positive, have also led to an increase demand by patients for even better outcomes and fewer complications (3, 4). Despite improvements in surgical technology and procedures, the risk of postoperative complications remains a significant challenge and strategies to mitigate this risk are active areas of investigation (5–7). Neurosurgeons are increasingly paying more attention to the influence of superior petrosal veins in the treatment of trigeminal neuralgia (8–12). However, there is less discussion about vein-related problems in MVD for hemifacial spasm (HFS). In MVD surgery for hemifacial spasm, in order to separate the responsible vessels in the root entry zone (REZ) of the facial nerve (FN), it is necessary to dissect the arachnoid membrane around the lower cranial nerves. During this procedure, we sometimes encounter IPV which obstructs the operation field. Although some experienced experts sacrifice IPV under this situation with no apparent postoperative complication (13, 14), it is reasonable to keep any of the vessels intact with the accomplishment of MVD surgery. Besides, the considerable variation of the inferior petrosal vein and its anatomic features have not been analyzed yet. Therefore, we made statistics on the diameter of IPV and also analyzed the obstruction of the surgical field by recording the location of IPV. We also provide a brief overview of the surgical technique applied for the management of these vessels.

## Methods

### Patients and Neuroimaging Analysis

From January 2014 to December 2020, 317 patients with HFS underwent MVD in the Department of Neurosurgery at Wuhan Union Hospital and Zhongnan Hospital of Wuhan University. All procedures were performed by a single neurosurgeon. Preoperative CT and/or MRI scans were performed on each patient to exclude secondary lesions such as intracranial tumors. MRI was performed using a 3.0 T scanner (Magnetom Trio; Siemens AG, Erlangen, Germany), and three-dimensional (3D) images were used for the preoperative evaluation. Non-contrast brain CT scans were also performed one day after MVD.

### Surgical Procedure

All patients underwent neurosurgery via retrosigmoid approach. After general anesthesia with endotracheal intubation, the patient was operated in the lateral position with the sagittal plane of the head parallel to the ground. With the horizontal reference plane, the patient’s head was lowered 15° and rotated to the contralateral side for about 15° without needing a head clamp. After being painted with antimicrobial solution, a 5 cm long incision was made into the skin and muscle layer in parallel with the hairline behind the ear. A mastoid spreader was used to widen the incision and expose a 2 cm × 2.5 cm bone window in the skull, and the posterior margin of the sigmoid sinus was exposed. The dura mater was incised with a microscope, and the cerebrospinal fluid was drained. After decreasing cerebellum tension, the cerebellopontine angle area and lower cranial nerves were exposed. The IPV was routinely located after dissecting the arachnoid membrane or sleeve. Typical intraoperative management was as follows: (1) Gentle retraction of the cerebellum to expose the root entry zone (REZ) of the facial nerve and confirm the responsible vessel; (2) Use of Teflon tape to transpose and fix the offending vessel; (3) Perform intraoperative neuro-electrophysiological monitoring (IONM) which in this procedure included Brainstem Auditory Evoked Potential (BAEP), Motor Evoked Potential (MEP), Somatosensory Evoked Potential (SEP), and Abnormal Motor Response (AMR).

### Intraoperative Management of the IPV

In most cases, the IPV was located near the lower cranial nerves where after passing through the arachnoid membrane, it drains into the jugular bulb. The arachnoid membrane and trabeculae are separated from IPVs to obtain a clear view of the surgical field. Protection of IPV is optimal if it can be located quickly to avoid venous tearing and bleeding, which is one of our primary surgical goals. We try to avoid unnecessary IPV sacrifice to the greatest possible extent and to preserve IPVs with a larger diameter. Admittedly, in many cases, MVD procedures cannot be completed without coagulation and cutting of small veins. To manage IPV rupture during the operation, we performed compression with a neurosurgical sponge or cottonoid to stop the bleeding. Once the hemorrhage is under control, the bleeding point is identified, and after coagulation with bipolar, the IPVs are cut.

### Postoperative Follow-up

After discharge, patients were required to visit our outpatient department every six months for follow-up observation. All recurring symptoms and complications were recorded in our electronic medical records. Follow-up ranged from 0.9–4 years in this cohort.

### Statistical Analysis

Chi-square test and Fisher exact test were adopted as appropriate, using SPSS (Version 22; SPSS Inc., Chicago, IL). *P*-values <0.05 were considered significant and values >0.05 not significant.

## Results

### Measurement of IPV and Intraoperative Observation

**Figure 1:**
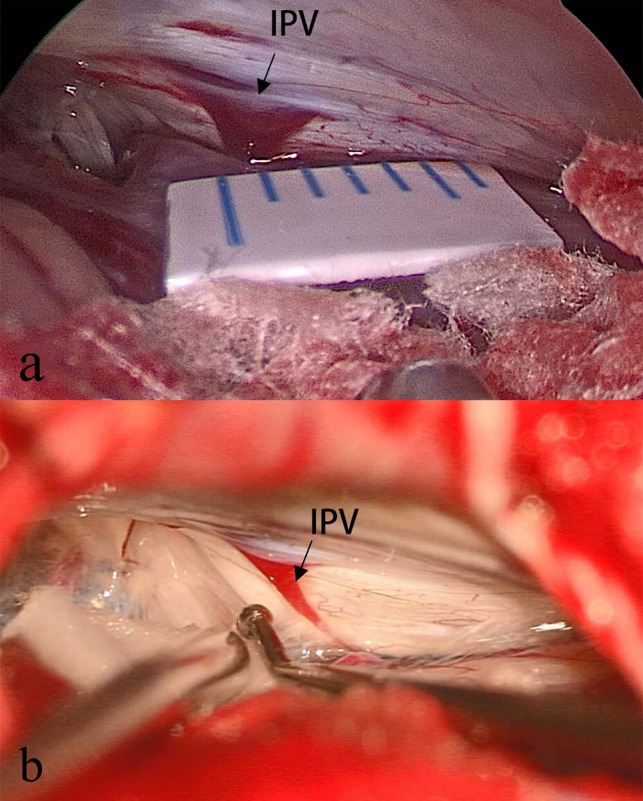
IPV location and intraoperative measurement. The IPV is always near the lower cranial nerves, and after passing through the arachnoid membrane, it drains into the jugular bulb. To measure IPV diameter, we use a ruler (**A**) placed within the operative area during the surgical procedure, and sometimes we use a vascular separator as reference (**B**).

**Figure 2:**
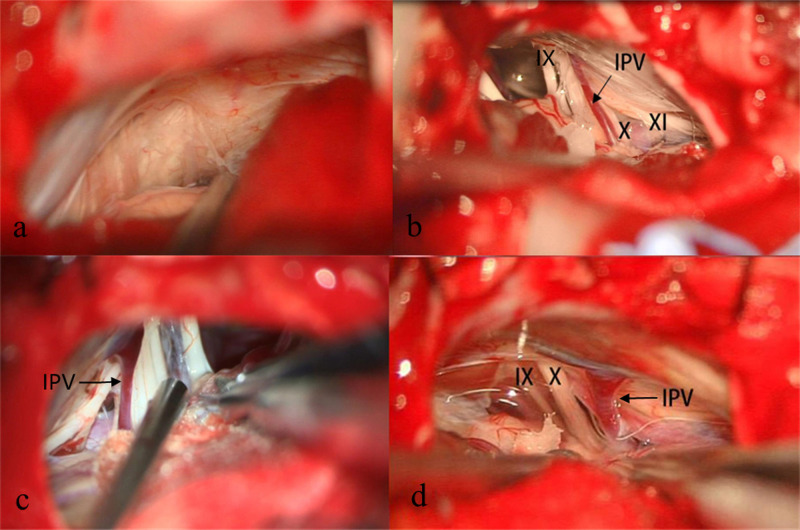
Example of patient with no apparent IPV (**A**). Three types of IPVs were recorded during MVD based on diameter- the diameter <0.5 mm (**B**), 0.5 mm–1 mm (**C**), >1 mm (**D**). IX, Glossopharyngeal Nerve; X, Vagus Nerve; XI, Accessory Nerve; IPV, Inferior Petrosal Vein.

**Figure 3:**
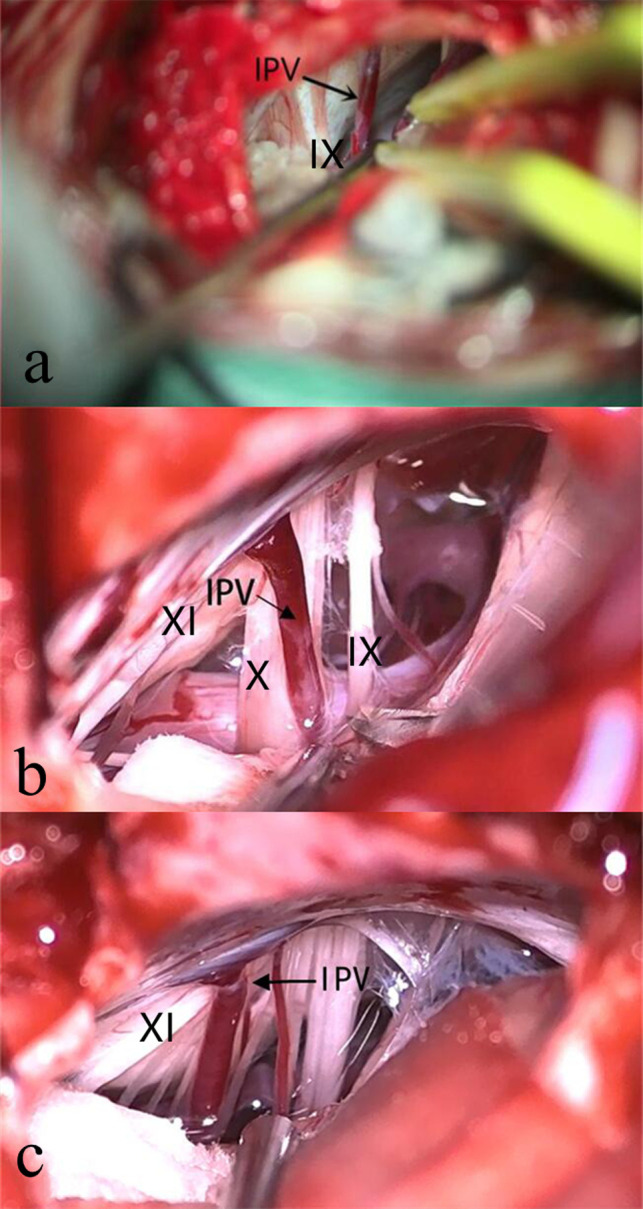
IPV Draining Points. The confluence of IPV and jugular bulb can be at or above the level of the glossopharyngeal nerve (**A**), at the level of the vagus nerve (**B**), or the level of the accessory nerve (**C**). IX, Glossopharyngeal Nerve; X, Vagus Nerve; XI, Accessory Nerve; IPV, Inferior Petrosal Vein.

IPVs were observed in 242 out of 317 cases. Patient characteristics and postoperative results are summarized in Table 1. The measurement of the diameter of IPV is performed at its middle cisternal portion, and we routinely use a scale or ruler for reference during the surgical procedure. (Figure 1A). If these are not available, the tip of a vascular separator or aspirator is placed alongside the IPV as a reference (Figure 1B), for the diameter of the tip of a vascular separator or suction is about 1 mm. Occasionally IPVs cannot be located (Figure 2A). Once IPVs are confirmed during procedure, they are divided into three types according to diameter: (1) <0.5 mm = 58 cases (Figure 2B); (2) 0.5–1.0 mm = 145 cases (Figure 2C); (3) >1 mm = 39 cases (Figure 2D). In the IPV sacrifice group, 38 cases have veins with diameters less than 0.5 mm, 66 cases have veins between 0.5 and 1 mm in diameter, and 6 cases have veins greater than 1 mm. In the IPV preservation group, 20 patients had vein diameters <0.5 mm, 79 patients between 0.5–1 mm, and 33 patients >1 mm. The preservation group had more veins larger than 1 mm in diameter, and the sacrifice group had more veins smaller than 1 mm (*P* < 0.01). The above statistical analysis is shown in Table 1.

**Table 1:** Patient characteristics.

Characteristics	Total (242)	IPVs sacrifice group (110)	IPVs preservation group (132)	*P-*value
Sex (M/F)	112/130	49/61	63/69	*P* = 0.62
Age, range, mean, yrs	32–68 (49)	38–65 (46)	32–68 (51)	*P* = 0.66
Disease duration, range, mean, yrs	0.5–20 (3.5)	2–15 (3.8)	0.5–20 (3.4)	*P* = 0.72
Outcome				*P* = 0.99
Symptom relief (immediate/delayed >2 weeks)	237/5	108/2	129/3	
Recurrence	7	3	4	*P* = 0.99
Complications				*P* = 0.32
Facial weakness	12	5	7	
Hearing impairment	10	6	4	
Cerebellar contusion	4	4	0	
Wound infection/CSF leakage	0	0	0	
Hemorrhage	0	0	0	
Hoarseness	8	4	4	
Irritated cough	8	3	5	
IPV Diameter				
<0.5 mm	58	38 (34.5%)	20 (15.2%)	*P* < 0.01
0.5 mm −1 mm	145	66 (60.0%)	79 (59.8%)	*P* = 0.98
>1 mm	39	6 (5.5%)	33 (25%)	*v* < 0.01
Level of IPV draining point				
Accessory nerve	163	56 (50.9%)	107 (81.1%)	*P* < 0.01
Vagus nerve	42	24 (21.8%)	18 (13.6%)	*P* = 0.09
Glossopharyngeal nerve	37	30 (27.3%)	7 (5.3%)	*P *< 0.01

Although the lower cranial nerves are close near the jugular foramen, the anatomical relationship during microvascular decompression is relatively stable. The course of glossopharyngeal nerve, vagus nerve, accessory nerve and the bridging veins of posterior fossa can still be identified during the operation. After locating the IPV, the shape is assessed with particular attention to the point where it merges into the jugular bulb. Based on the confluence patterns, the IPVs are divided into three types according to the draining point: Type (1) draining at or above the level of the glossopharyngeal nerve (37 cases, Figure 3A); Type (2) draining at the level of the vagus nerve (42 cases, Figure 3B); Type (3) draining at the level of the accessory nerve (163 cases, Figure 3C). In the sacrifice group, 56 cases have draining points at the level of the accessory nerve, 24 cases at the vagus nerve level, and 30 cases at the level of or above the glossopharyngeal nerve. In the preservation group, the draining point is at the level of the accessory nerve in 107 cases, the vagus nerve in 18 cases, and the glossopharyngeal nerve in 7 cases. The draining points of IPV in the sacrifice group were mainly at the level of the glossopharyngeal nerve, while in the preservation group were mainly near the accessory nerve (*P* < 0.01). The above statistical analysis is shown in Table 1.

### Sacrifice or Preservation Status

Electrocoagulation was used to mitigate IPV tearing or hemorrhage in 110 of 242 cases where IPVs were located. Most of our intraoperative IPV injuries occur in vessels with a diameter <1 mm, and in some cases, even the action of gentle pulling on the cerebellum may lead to laceration of the vein. Deliberate injury by the operator is not the leading cause of nearly half of IPV vein sacrifices. Four of the cases with IPV diameter >1 mm had intraoperative hemorrhage. After hemostasis by compression, the IPVs were handled by electrocoagulation when bleeding was mild or continued to be compressed by a neurosurgical sponge until there was no active hemorrhage. Mitigation of bleeding is routinely confirmed by postoperative CT scans. In 132 cases where IPVs were located, the veins were preserved during the operation. Importantly, the IPVs in preservation group were more likely to be larger in diameter >1 mm (25% vs. 5.5%, *P* < 0.01) and more likely to be located at the accessory nerve level (81.8% vs. 50.9%, *P* < 0.01). The diameters of IPV in the sacrifice group were mainly less than 1 mm (94.5% vs. 75%, *P* < 0.01), and the cases with draining points near the glossopharyngeal nerve were more than that in the preservation group (27.3% vs. 5.3%, *P* < 0.01).

### Postoperative Complications

Twelve patients experienced mild postoperative facial paralysis but all recovered within six months. Ten cases presented with postoperative hearing impairment based on pure tone average threshold and speech discrimination score tests. According to outpatient clinic testing and telephone follow-ups, most of the ten patients’ hearing function were restored to preoperative state, while one patient improved compared to postoperative tests. Meanwhile, no permanent postoperative hearing loss was observed in this study. Hoarseness and an irritated cough when drinking water was observed in 8 cases. No lingering postoperative wounds or intracranial infections were observed in our study. Other complications, such as postoperative CSF leakages and hemorrhage were not observed either.

Six cases of IPVs with a diameter >1 mm were sacrificed during the operation, but no postoperative hemorrhage was observed. Of these six patients, four had cerebellar contusions, which were found by postoperative CT scan to manifest as hypointense lesions in the cerebellum. This contusion determination was made based on intraoperative observation and postoperative review of the surgical video, which clearly identified the location of postoperative hypodense lesion on CT scan was the same location as excessive traction and mild injury to the cerebellum. None of these patients had any new nervous system dysfunction. Every IPV rupture was controlled with proper management as outlined above. The surgical outcomes and follow-up results showed no significant difference between the IPV sacrifice and preservation groups.

### Surgical Efficacy

All patients with hemifacial spasm had efficacy judgment for at least one year. Seven patients relapsed, and five showed marked improvement with only a few facial tics observed. Two patients underwent a redo operation because of ineffective initial surgery, and were subsequently cured. No significant differences were observed in long-term efficacy between the IPV sacrifice and preservation groups.

## Discussion

Classical definition of the IPV refers to the bridging vein entering the inferior petrosal sinus (IPS) (15). However, similar to the superior petrosal vein, the IPV may also be composed of a group of small veins (16) that coursed anteriorly to the lower cranial nerves and emptied to the IPS, or may enter the jugular bulb between or behind the lower cranial nerves (17). Our primary concern is with the bridging veins behind the lower cranial nerves and entering the jugular venous bulb, as they may have an impact on the exposure of the REZ for microvascular decompression procedure. In MVD surgery for HFS, to expose the responsible blood vessels, it is necessary to separate the arachnoid around lower cranial nerves (the vagus nerve, accessory nerve, and especially glossopharyngeal nerve) (18). Because the IPV courses through the superficial surface of the lower cranial nerves, the locations of these veins are likely to affect the surgical area (13, 19). Our research found that the degree of variation in IPV diameter and location is considerable, and there is a paucity of studies on related anatomic features. Therefore, it is meaningful to discuss the two aspects of IPV variation and their impact on MVD procedure.

### Perspectives on IPV Diameter

Though IPV diameter varies greatly among patients, in most cases of the sacrifice group, the diameters were generally smaller (i.e., <1 mm). There are two plausible reasons for this- first, a smaller IPV is difficult to protect because of its thin and fragile wall, which is more prone to tearing; second, sometimes experienced surgeons opt to deliberately sacrifice an IPV with small diameters in order to achieve wider exploration (13, 14). A small diameter indicates that the draining area of the IPV is more limited, meaning that any injury to the vessel is likely to have little effect on the patient. No severe postoperative neurological dysfunction was observed in the IPV sacrifice group, and this confirms our hypothesis about the diminished effect of tearing in the small IPV cases. As for larger IPV diameter cases, it is reasonable to believe that there is more risk involved in damaging the IPV because of the larger drainage area (20). This phenomenon is intuitive and generalized for all large vein ruptures, especially when bleeding is typically more severe. Although there are no cases of postoperative hemorrhage and cerebellar infarction after IPV damage, considering that we have adopted an individualized strategy and included a limited sample size in sacrifice group with IPV >1 mm, it is reasonable to protect vessels >1 mm. Most of reported findings about IPV sacrifice are based on the experience of the surgeon for decision-making. Our research may provide intraoperative images and detailed data as reference for other scholars.

### Perspectives on IPV Draining Point in Jugular Bulb

We have observed that the IPV might enter the jugular bulb (JB) at different anatomic locations. Although most of the IPVs enter the JB alongside the accessory nerve, we still find some of them drain into sites near the vagus nerve or glossopharyngeal nerve. Whether this change has a specific anatomical significance has not been reported. We believe that if the confluence point of the IPV is near the glossopharyngeal nerve, it means that the trunk of IPV is closer to the REZ of the facial nerve, which will significantly affect the exposure of the responsible blood vessels. If the confluence point is near the vagus nerve or the accessory nerve, the trunk of the IPV is away from the REZ of the facial nerve. Compared with the preservation group, there were more cases with IPV at the level of the glossopharyngeal nerve in the sacrifice group (*P* < 0.01). These results corroborate our knowledge that IPV near the glossopharyngeal nerve has a greater impact on REZ exposure. Different confluence points are aspects of the IPV variation. Therefore, it is necessary to pay more attention and adopt surgical strategies according to variable situations during the procedure. The more complicated problem is to decide which factor comes first for intraoperative decision-making, the draining point or diameter. There is no statistical significance with complication rates between the two groups, which is consistent with the acknowledgment of other experienced experts. Therefore, acquiring a better view of the operation field by sacrificing IPV near the glossopharyngeal nerve is reasonable. However, considering there were only 6 cases in the sacrifice group with a diameter >1 mm, the statistical result may need to be interpreted cautiously under this sophisticated situation. Nevertheless, it is still feasible to protect IPV with a diameter >1 mm through a complete dissection of the arachnoid membrane to avoid unknown consequences as much as possible.

### Perspectives on the Intraoperative Management of IPV

To protect the IPV, our experience informs us that it must first be fully exposed. Therefore, we emphasize thorough dissection of the arachnoid membrane and complete removal of adhesions between the IPV and lower cranial nerves. After the IPV is torn, bleeding may be initially turbulent, especially when the breach is located where the vein injects into the jugular bulb. IPV rupture can have two adverse consequences: (1) arbitrary electrocoagulation and heat conduction may injure the lower cranial nerves; (2) hemorrhaging blood can penetrate the arachnoid membrane, making it challenging to identify relevant anatomic structures and risking further damage. If the IPV ruptures, we suggest a more limited and targeted use of electrocoagulation. Another meaningful discussion of intraoperative management is about the method for retraction. Our procedures are all performed with dynamic retraction, mainly using suction and vascular separators. Because the diameter of IPV is often small, dynamic retraction can help observe the morphological change of IPV when it is stretched. The surgeon can adjust the manipulation strength accordingly to avoid accidental IPV rupture. In short, the exposure and assurance of surgical efficacy are the first priority, and on this basis, efforts can be appropriately focused on the protection of the IPVs, especially those with larger diameter larger (>1 mm).

### Limitations

Our study does have several limitations which merit discussion. First, the incidence of cerebellar contusion in the two groups were low. Even in the IPV sacrifice group, this incidence was too small relative to the overall sample size to discern a significant effect. Therefore, a similar study with larger sample size is needed to sufficiently elucidate the effect of IPV sacrifice on contusion. Second, we encountered just 6 cases where there was an IPV impairment with >1 mm diameter. Whether it is safe to sacrifice in such a situation warrants further study. We clarified that the case of cerebellar contusion was related to exposure, and our intraoperative observation and postoperative review of the surgical video clearly identified the occurrence of excessive traction and mild injury to the cerebellum. However, venous injury may still be a factor in the occurrence of postoperative CT hypodensity lesions. Based on these observations, more single-factor cases are needed to further distinguish the cause. Finally, the method used to assess IPV diameter needs improvements because accurately gauging the diameter in patients during surgery is difficult (as compared with cadaveric specimen). However, it is our experience that intraoperative measurements using simple scales and surgical instruments can assist the operator with intraoperative decision-making and improve outcomes. Due to concerns that additional vascular tests such as DSA and CTA would impose a financial and physical burden on patients, these tests specifically for IPV were not adopted. 3D-CISS sequences, T1 and T2 images are the most common way to assess the responsible vessels with surrounding nerves for hemifacial spasm. Omission of veins below 1 mm in diameter in MRI frequently happens (21). The boundary resolution was 0.58–0.8 mm in vessel diameter even with the improved protocol (22). However, the diameters of IPVs are mostly less than 1 mm, and even some are less than 0.5 mm. Hence, there are difficulties in preoperative identification. Besides, on 3D-CISS images, the signal of IPV is close to that of the facial nerve, so it is difficult to distinguish the two. Perhaps a combination of preoperative assessment using a modified magnetic resonance technique with intraoperative validation using the method in our study could help to improve the accuracy of IPV measurement.

## Conclusion

The objective of our study was not to validate a specific surgical strategy, rather to present IPV variation, anatomic features and impact of these findings on surgical procedure. IPV is an obstructive structure in MVD for HFS, with considerable variations in diameters and draining points. IPV near the glossopharyngeal nerve significantly impacts surgical exposure and is often sacrificed for a better view of the operation field. Meanwhile, it is feasible to preserve IPV with a diameter >1 mm.

## Data Availability

The raw data supporting the conclusions of this article will be made available by the authors, without undue reservation.
